# Changes in the quality of cause-of-death statistics in Brazil: garbage codes among registered deaths in 1996–2016

**DOI:** 10.1186/s12963-020-00221-4

**Published:** 2020-09-30

**Authors:** Elisabeth França, Lenice Harumi Ishitani, Renato Teixeira, Bruce B. Duncan, Fatima Marinho, Mohsen Naghavi

**Affiliations:** 1 Programa de Pós-Graduação em Saúde Pública, Faculdade de Medicina, Universidade Federal de Minas Gerais, Av. Prof. Alfredo Balena, 190, sala 731, Santa Efigênia, Belo Horizonte, MG 30130-100 Brazil; 2 Research Group in Epidemiology and Health Evaluation, Universidade Federal de Minas Gerais, Av. Prof. Alfredo Balena, 190, Belo Horizonte, 30130-100 Brazil; 3 Programa de Pós-graduação em Epidemiologia, Universidade Federal do Rio Grande do Sul, R. Ramiro Barcelos 2600/414, Porto Alegre, 90035-003 Brazil; 4 Vital Strategies, 61 Broadway, Suite, New York, NY 1010 USA; 5 Institute for Health Metrics and Evaluation, 2301 5th Avenue, Suite 600 Box 358210, Seattle, WA 98121 USA

**Keywords:** Cause of death, Data quality, Vital statistics, Brazil

## Abstract

**Background:**

Registered causes in vital statistics classified as garbage codes (GC) are considered indicators of quality of cause-of-death data. Our aim was to describe temporal changes in this quality in Brazil, and the leading GCs according to levels assembled for the Global Burden of Disease (GBD) study. We also assessed socioeconomic differences in the burden of different levels of GCs at a regional level.

**Methods:**

We extracted data from the Brazilian Mortality Information System from 1996 to 2016. All three- and four-digit ICD-10 codes considered GC were selected and classified into four categories, according to the GBD study proposal. GC levels 1 and 2 are the most damaging unusable codes, or major GCs. Proportionate distribution of deaths by GC levels according selected variables were performed. Age-standardized mortality rates after correction of underreporting of deaths were calculated to investigate temporal relationships as was the linear association adjusted for completeness between GC rates in states and the Sociodemographic Index (SDI) from the GBD study, for 1996–2005 and 2006–2016. We classified Brazilian states into three classes of development by applying tertiles cutoffs in the SDI state-level estimates.

**Results:**

Age-standardized mortality rates due to GCs in Brazil decreased from 1996 to 2016, particularly level 1 GCs. The most important GC groups were ill-defined causes (level 1) in 1996, and pneumonia unspecified (level 4) in 2016. At state level, there was a significant inverse association between SDI and the rate of level 1–2 GCs in 1996–2005, but both SDI and completeness had a non-expected significant direct association with levels 3–4. In 2006–2016, states with higher SDIs tended to have lower rates of all types of GCs. Mortality rates due to major GCs decreased in all three SDI classes in 1996–2016, but GC levels 3–4 decreased only in the high SDI category. States classified in the low or medium SDI groups were responsible for the most important decline of major GCs.

**Conclusion:**

Occurrence of major GCs are associated with socioeconomic determinants over time in Brazil. Their reduction with decreasing disparity in rates between socioeconomic groups indicates progress in reducing inequalities and strengthening cause-of-death statistics in the country.

## Background

Analyses of causes of death (COD) are fundamental for monitoring the health situation of populations in order to better inform policy decisions about priority health interventions [1, 2]. Brazil has more than 1 million deaths registered with listed causes each year in the Mortality Information System (SIM in Portuguese). All causes related to the death should be declared in the standard death certificate (DC) used. The registration of a well-defined underlying cause that triggered the chain of events that led to the death is fundamental for public health interventions, as mortality statistics are produced with a single cause for each death which allows interventions that target this basic cause. Inadequate or insufficiently specified COD statements can compromise the reliability of these statistics and their proper use by policy-makers to plan suitable interventions [1].

The traditional approach to assess the accuracy of mortality information is to measure completeness (percent of all deaths registered), and the proportion of total deaths coded to R codes from the ICD 10-chapter 18 of general signs and symptoms, denominated as ill-defined causes [1, 3]. Death registration completeness in Brazil is estimated to be high in recent years, more than 95% in the period 2000–2010, although with large regional differences. On the other hand, quality of cause-of-death data on registered deaths remains a problem, as the country presented high levels of ill-defined causes during the last decade [4]. A national official intervention by the Ministry of Health (MoH) was started in 2005 to investigate ill-defined causes of death in health facilities and at home in the whole country, particularly in the poorest North and Northeast regions [5]. Reported results indicate that this investigation has been effective [6].

In addition to R codes from the ICD 10-chapter 18, other ill-defined or unspecified causes that do not represent useful underlying causes from a policy perspective were analyzed in the 1990 Global Burden of Disease (GBD) Study by Murray and Lopez (1996) [7], who introduced the term garbage codes (GC) for those causes. In 2010, an innovative approach was proposed by Naghavi and colleagues which greatly expanded the number of codes to be considered garbage, including several ill-defined and incomplete codes for cardiovascular diseases, neoplasms, injuries, and in other ICD chapters. These causes were classified according to four groups in a proposed typology. To enhance comparability of COD data, they selected most probable target codes and proposed algorithms for GC redistribution to the more appropriate target codes [8]. This approach was updated in the 2010, 2013, and 2015 posterior publications of Global Burden of Disease (GBD) studies.

The GBD 2016 study presented a new classification of GCs in four categories, according to the levels of the GBD cause list across which they can be redistributed [9]. This classification was also used in the subsequent GBD 2017 study. It classifies codes that the correction of which causes the new code to fall into a different 1st level GBD cause group as level 1. Other GCs whose correction has impact at subsequent levels of cause are categorized, on the basis of the GBD cause level impacted by the change, as levels 2, 3, and 4. Level 1 and 2 GCs are considered major GCs and their fractions used in a composite metric to rate the quality of mortality data available for each country [10]. As this new proposed classification is based on the extent of redistribution of GCs across the overall COD distribution, it actually provides an important improvement in the analysis of quality of mortality data.

In this study, we describe temporal changes in the quality of vital statistics in Brazil, and the leading GCs according to levels assembled for the Global Burden of Disease (GBD) study. Some selected variables are also reported due to their relevance for health policy-makers to facilitate adequate interventions. We also assess socioeconomic differences in the burden of different levels of GCs at a regional level.

## Methods

### Overview

A descriptive data analysis of GCs was conducted based on all deaths registered in the SIM from 1996 to 2016 for both sexes and all ages. There are mandatory laws for the state to register deaths at civil registry offices, and this information is compiled by the Institute of Geography and Statistics (IBGE). Since 1975, the MoH has created the SIM to complement the work of IBGE in vital statistics, with responsibility for collecting and processing mortality data across the whole country, in particular cause of death data. Municipal teams are responsible for the active recording of deaths in hospitals and collection of home deaths in civil registry offices. Standard forms of DCs constitute the source of information of this system and should be filled in by physicians in accordance with the WHO guidelines. The International Classification of Diseases (ICD) coding rules are used to select the underlying COD through an automated coding system named SCB (Seletor de Causa Básica-Underlying COD Selector) [11].

We selected all three- and four-digit ICD-10 codes considered GC according to the GBD 2017 study, defined as a cause that cannot be considered an underlying cause of death or is not well specified. These codes were classified into four levels [10], according the redistribution they generated with respect to the proposed redistribution into the GBD hierarchical cause list. Thus, level 1 GC such as the majority of ill-defined causes from chapter 18-ICD10 or septicemia could have as underlying cause an injury, a non-communicable disease, or an infectious disease, and could potentially be redistributed to any level 1 group within GBD cause hierarchy. Level 2 GCs include all codes that are redistributed to within one of these three broad groups but to different level 2 GBD cause. Examples of these GCs are injury unspecified, essential hypertension, and pulmonary edema. Level 3 and level 4 GC codes comprise those that can potentially be redistributed to different level 3 and level 4 causes of the GBD cause hierarchy, respectively. An example of a level 3 GC is cancer unspecified, which contains sufficient detail to be redistributed across all types of cancer from the level 3 GBD cause list, but not outside that group. Unspecified stroke or unspecified road injuries are examples of GC level 4. It should be highlighted that R codes, which were traditionally named ill-defined causes of death, have been broken down into level 1 and level 2 GCs in our analysis, herein named R-codes level 1 and R-codes level 2, and a few R codes were not classified as GCs (R50.2, R78.0–R78.5. R95). The complete list of ICD codes classified as GCs in the GBD 2017 study according the four levels [12] is presented in Supplementary file 1.

We identified the number of deaths in the SIM that have been assigned to each level per year, and the leading GCs by sex in the first and last year of the period analyzed, 1996 and 2016. Proportionate distribution of deaths by GC levels and selected variables (age, place of death, and category of certifying physician) were also calculated in the most recent year to address the effect that ill-defined codes could have on more current health policy priorities.

### Division of Brazil into states and regions

Brazil has an estimated total population in 2016 of about 209 million divided into 26 states and a Federal District. Those geographies, hereafter referred to as states, are grouped into five regions:

1. North region: Composed of seven states (Acre, Amapá, Amazonas, Pará, Rondônia, Roraima, and Tocantins)—17.7 million inhabitants (8.6% of total population); responsible for almost half of the Brazilian territorial extension; and has the lowest population density of the country with living standards below the national average
2. Northeast: Composed of nine states (Alagoas, Bahia, Ceará, Maranhão, Paraíba, Pernambuco, Piauí, Rio Grande do Norte, and Sergipe)—56.9 million inhabitants (28% of the total population) with the lowest levels in almost all of the social indicators
3. Southeast: Composed of four states (Espírito Santo, Minas Gerais, Rio de Janeiro, and São Paulo)—86.3 million inhabitants (42% of the total population); responsible for almost 50% of the Brazilian GDP
4. South: Composed of three states (Paraná, Rio Grande do Sul and Santa Catarina)—29.4 million inhabitants, and higher living standards
5. Center West: Composed of the Federal District and three states (Goiás, Mato Grosso and Mato Grosso do Sul)—15.6 million inhabitants (6% of the total population) with intermediate living standards

### Analysis of temporal changes of garbage codes in states and by level of development based on the SDI

Mortality rates of GCs were calculated after correction of underreporting of deaths in the SIM. Estimated GC numbers by level were calculated by applying the proportions of GCs in each level from the SIM database to all-cause mortality estimated by the GBD 2017 study in each state. Age-standardized mortality rates, per 100,000, were calculated by the direct method using the GBD world’s population as reference [12]. Population data for age, sex, and states were obtained from the IHME [13]. For visualizing the geographical distribution of GC rates between 1996 and 2016 at the state level, we aggregated GC levels 1 and 2, considered major GCs [9, 10]. Similarly, GC levels 3 and 4 were summed up.

We investigate the temporal relationship between level 1–2 and 3–4 GC rates in states by the Sociodemographic Index (SDI), a composite measure proposed in the GBD study based on the average total fertility under the age of 25 years, educational attainment in those aged 15 years or older, and lag distributed income per capita. Each indicator was scaled to a value from zero to one, and the composite SDI index is the mean of the 3 rescaled components. This index varies between zero and one, with zero representing the lowest per capita income, lowest level of education and highest total fertility rate under the age of 25 years, i.e., the location with a theoretical minimum level of development relevant to health. An SDI of 1 represents the highest per capita income, the highest level of education and lowest total fertility under the age of 25 years, this location having a theoretical maximum level of development [10].

The intervention of the MoH implemented in Brazilian states in 2005—the investigation of GC level 1 R-codes, responsible for a higher proportion of GCs in the country (14.3% out of 946,960 deaths recorded at SIM in 2000), impacted on reducing those codes and improved the quality of cause of death data [6]. To control for the effect of this confounding factor in time series analysis of socioeconomic differences, we considered two separate periods in this analysis, 1996–2005 and 2006–2016. Linear regression coefficients were separately modeled in these two periods to examine the linear association between GC mortality rates of each type and SDI and how this association changed after the intervention. Although we controlled for the influence of completeness using mortality rates adjusted for undercount of deaths, we also examined whether this variable was associated with the occurrence of GC levels 1–2 and 3–4 in Brazilian states for each period analyzed.

To better visualize the effect of differences developmental on level 1–2 and 3–4 GC rates at a regional level during the whole analyzed period, we classified states into tertiles of SDI state-level estimates for the first year of analysis, 1996, as follows: (1) low SDI, states with SDI values lower than 0.460—Acre, Alagoas, Bahia, Ceará, Maranhão, Paraiba, Pará, Piauí, and Tocantins; (2) medium SDI, states with SDI values varying from 0.460 to 0.540—Amapá, Amazonas, Goiás, Mato Grosso do Sul, Pernambuco, Rio Grande do Norte, Rondônia, Roraima, and Sergipe; and (3) high SDI: states with SDI values higher than 0.540—Distrito Federal, Espírito Santo, Mato Grosso, Minas Gerais, Paraná, Rio Grande do Sul, Rio de Janeiro, Santa Catarina, and São Paulo.

States in the North and Northeast regions were classified in either the low or middle SDI groups, while all states pertaining to the South or Southeast regions were in the high SDI category.

## Results

**Fig. 1:**
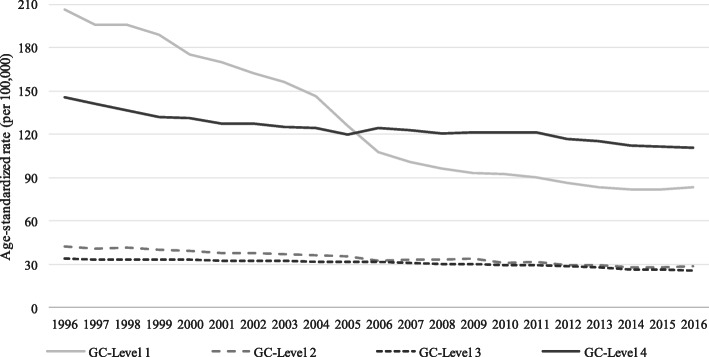
Age-standardized mortality rates from garbage codes according to levels. Brazil, 1996–2016

Age-standardized mortality rates due to deaths coded to GCs registered as COD in Brazil decreased from 417.3 per 100,000 inhabitants in 1996 to 261.3 in 2016. This decrease occurred particularly in the GC level 1 group, which has been the most important group of GCs from 1996 to 2005, but was overtaken by GC level 4 in the latter years (Fig. 1).

Table 1 presents the proportional mortality distribution by sex according to levels and the leading GC groups in the country, in 1996 and 2016. In 1996, 45.3% and 52.5% of registered deaths were classified as GCs in males and females, respectively. These proportions decreased to 36.0% and 42.2% of all deaths in 2016, and the proportions of deaths coded to level 1 GCs dropped in those years from 20.7 to 12.5% in males, and from 25.1 to 14.0% in females. Although GCs were still coded for approximately 507,000 deaths (both sexes) in 2016, levels 3 and 4 were more frequent in that year.

**Table 1:** Leading garbage codes in males and females. Brazil, 1996 and 2016

**Level**	**Cause of death (ICD code**^**a**^**)**	**1996**				**2016**			
		**Male**		**Female**		**Male**		**Female**	
		***n***	**%**	***n***	**%**	***n***	**%**	***n***	**%**
4	Pneumonia unspecified (J15.9, J18, J22)	18350	3.5	15330	4.1	40227	5.5	41020	7.2
4	Stroke, not specified as hemorrhage or infarction (I64, I67.8–I67.9, I69.4, I69.8)	29087	5.5	27537	7.3	37219	5.1	35878	6.3
1	Ill-defined causes—R codes (R02–R99—level 1)	70164	13.2	57260	15.2	39447	5.4	28922	5.1
4	Diabetes mellitus, unspecified	10263	1.9	14993	4.0	22928	3.1	27955	4.9
1	Heart failure (I50)	15880	3.0	17217	4.6	13798	1.9	14976	2.6
2	Essential hypertension (I10)	2934	0.6	3697	1.0	11783	1.6	13352	2.3
3	Cancer, unspecified site (C14, C26, C39, C46, C55, C579, C639, C689, C759, C76, C77, C78, C79, C80, C97)	5737	1.1	7718	2.0	10229	1.4	11234	2.0
1	Septicemia (A40–A41)	5776	1.1	5095	1.4	9769	1.3	10091	1.8
4	Dilated and unspecified cardiomyopathy (I42.0, I42.9)	5951	1.1	4938	1.3	5857	0.8	4268	0.7
2	Injury, undetermined intent (Y20–Y34)	7658	1.4	1615	0.4	7336	1.0	2396	0.4
1	Acute renal failure and unspecified kidney failure (N17, N19)	2499	0.5	1951	0.5	4326	0.6	3636	0.6
1	Pulmonary embolism (I26)	1962	0.4	2230	0.6	3118	0.4	4292	0.7
3	Other respiratory disorders (J98.0, J98.4–J98.9)	3547	0.7	3187	0.8	3579	0.5	3733	0.7
2	Ill-defined causes—R codes (R02–R99—level 2)	4751	0.9	4091	1.1	3829	0.5	3287	0.6
4	Transport accident, type of vehicle unspecified (V89)	13033	2.5	3415	0.9	5850	0.8	1159	0.2
2	Hematemesis, melena, intestinal hemorrhage, unspecified (K92.0–K92.2)	1709	0.3	1239	0.3	3585	0.5	2762	0.5
1	Respiratory failure (J96)	3272	0.6	3153	0.8	2363	0.3	2364	0.4
1	Pneumonitis due to solids and liquids (J69)	699	0.1	621	0.2	2384	0.3	2294	0.4
3	Perinatal period condition, unspecified	770	0.1	544	0.1	266	0.0	217	0.0
1,2,3,4	Other garbage codes	35909	6.8	21880	5.8	37588	5.1	27561	4.8
	GC total	239951	45.3	197711	52.5	265481	36.0	241397	42.2
	Level 1	109953	20.7	94643	25.1	92102	12.5	79980	14.0
	Level 2	30234	5.7	18535	4.9	33076	4.5	27394	4.8
	Level 3	15975	3.0	16127	4.3	24323	3.3	22530	3.9
	Level 4	83789	15.8	68406	18.2	115980	15.7	111493	19.5
	Non-GC	290118	54.7	178791	47.5	471361	64.0	330962	57.8
	All causes-total	530069	100.0	376502	100.0	736842	100.0	572359	100.0

^a^When only the 3-digit code is specified it includes all 4-digit codes

The top five ICD-10 grouping codes of COD classified as GCs in males and females in 2016 were (a) pneumonia unspecified (level 4 classification in the GBD study), (b) stroke not specified as hemorrhage or infarction (level 4), (c) ill-defined causes (level 1), (d) diabetes unspecified (level 4), and (e) heart failure (level 1). Proportions of the majority of leading GC groups showed in Table 1 decreased or maintained relatively stable in 2016 compared to 1996. R-codes level 1 decreased considerably over the same period in males and females. On the other hand, proportions of the level 4 GCs acute lower respiratory infections and unspecified diabetes and the level 2 GC essential hypertension all increased 1 percentage point or more in 2016.

Table 2 shows the percentage of GC and non-GC coded deaths in both sexes combined by age, place of death, and certifying physician in 2016. The age groups with the highest proportion of GCs were the elderly—over 60 years of age, and especially over 80 years (50%), followed by children aged 1–9 years (greater than 34%). While among those under 50 years old GC level 1 predominate, among the elderly (over 60 years) GC level 1 is exceeded by GC level 4. Concerning the place of death, more than 70% of total deaths in the country in 2016 occurred in hospitals. Garbage codes were present as the underlying cause of death in 46% of home deaths and in 38% of hospital deaths. Higher proportions of major GC levels 1–2 were present in deaths occurring at home. On the other hand, level 4 GCs were more frequent for in-hospital deaths. As expected, DCs signed by coroners from forensic institutes and physicians from the autopsy service for natural causes (herein named SVO physicians), had lower proportions of GCs, although more than 35% of these deaths were declared as being due to GCs. GC fractions from the attending and substitute physicians were almost 40%, with slight differences between them.

**Table 2:** Deaths classified as garbage codes (GC) according to selected variables. Brazil, 2016

**Variable**	Garbage codes					Non-GC (%)^a^	Total deaths (%)^b^
	Level 1 (%)^a^	Level 2 (%)^a^	Level 3 (%)^a^	Level 4 (%)^a^	Total GC (%)^a^		
**Age**							
< 01	6.0	0.9	3.0	4.2	14.2	85.8	36350 (2.8)
1–4	20.2	3.4	2.6	12.9	39.1	60.9	6212 (0.5)
5–9	18.7	3.4	2.5	9.6	34.2	65.8	3297 (0.3)
10–14	16.9	3.6	2.0	7.7	30.3	69.7	4877 (0.4)
15–19	8.2	4.1	0.8	6.5	19.6	80.4	21788 (1.7)
20–29	8.0	4.3	1.2	7.4	20.8	79.2	55643 (4.2)
30–39	10.6	4.3	2.4	8.8	26.0	74.0	64864 (5.0)
40–49	12.1	4.4	3.7	10.9	31.1	68.9	92650 (7.1)
50–59	12.1	4.2	4.4	13.2	33.8	66.2	157797 (12.0)
60–69	12.0	4.2	4.2	16.7	37.1	62.9	221752 (16.9)
70–79	13.1	4.5	3.8	20.7	42.1	57.9	265220 (20.2)
80 e+	16.5	5.7	3.6	23.9	49.6	50.4	376145 (28.7)
Missing	23.6	11.2	1.4	12.2	48.4	51.6	3179 (0.2)
**Place of death**^**a**^							
Hospital/health facility	11.4	3.7	4.0	19.2	38.3	61.7	948085 (72.4)
Home	21.3	7.5	3.2	14.1	46.0	54.0	256134 (19.6)
Street	5.2	4.4	0.3	9.3	19.2	80.8	59974 (4.6)
Others	13.2	7.2	1.4	9.4	31.3	68.7	44110 (3.4)
Missing	24.0	8.6	2.1	10.3	45.1	54.9	1471 (0.1)
**Who signed the original DC**							
Attending physician	12.6	4.0	4.0	19.3	39.9	60.1	343891 (26.3)
Substitute physician	12.4	3.4	4.4	19.3	39.4	60.6	293661 (22.4)
Forensic institute physician	12.4	14.9	0.7	8.1	36.1	63.9	175410 (13.4)
SVO physician^c^	14.4	3.8	2.9	15.0	36.1	63.9	86830 (6.6)
Other physician	18.2	5.1	4.2	18.0	45.5	54.5	271228 (20.7)
Missing	26.2	5.4	3.6	17.2	52.5	47.5	138754 (10.6)

^a^Fractions of each row total

^b^Fractions of all deaths (*n* = 1309774)

^c^SVO physician = physician from the Death Verification Service.

Supplementary file 2 presents the previous variables in males and in females. A higher percentage of GC levels 1 and 4 in women than in men for deaths occurring on streets and certified by coroners is probably due to the observed higher mortality due to injuries in men and better certification by forensic institute physicians. Deaths in women occurring on streets or certified by forensic institutes have higher probability of so-called natural causes and were certified as with garbage codes. The higher proportion of GCs in the age group of 15 to 29 years in women might be related to this hypothesis.

**Fig. 2:**
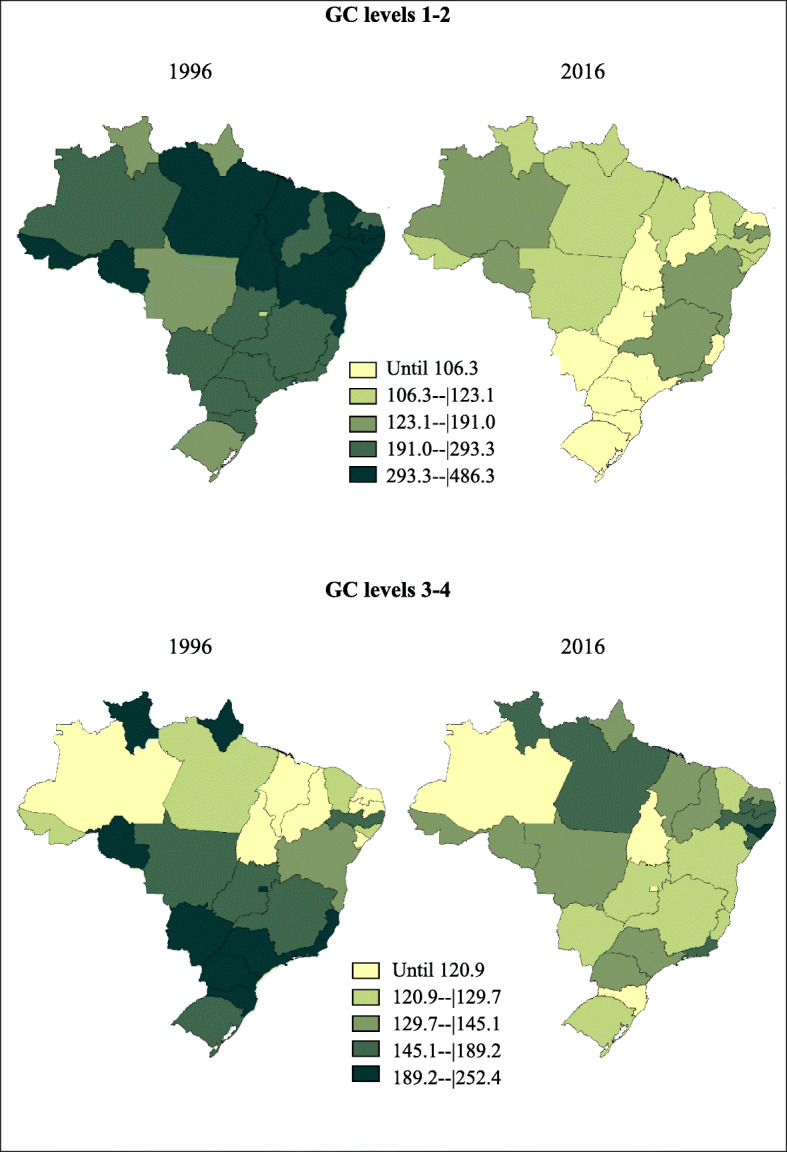
Age-standardized mortality rates from deaths coded to level 1 or 2 garbage codes (above) and to level 3 or 4 codes (below) in Brazilian states in 1996 (right panel) and 2016 (left panel)

Figure 2 shows age-standardized mortality rates for deaths coded to garbage codes in Brazilian states, in 1996 and 2016. All Brazilian states had lower rates of GC levels 1–2 in 2016 compared to 1996. Interestingly, for GC levels 3–4, several states from the North and Northeast regions presented higher rates in 2016. On the other hand, all states from the South and Southeast presented lower GC levels 3–4 rates in 2016.

**Fig. 3:**
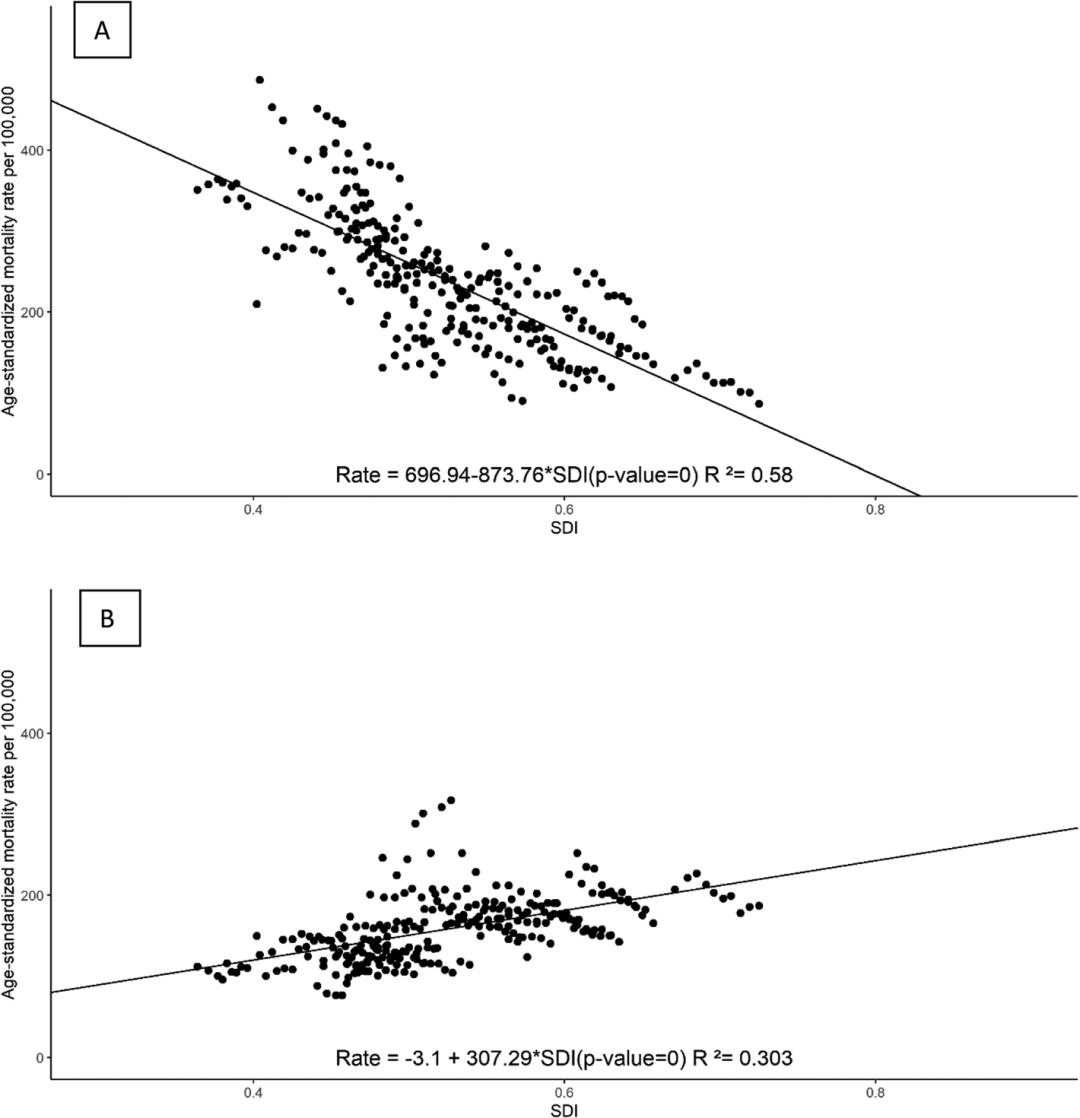
Age-standardized mortality rates from deaths coded to level 1 or 2 garbage codes (**a**) and to level 3 or 4 garbage codes (**b**) by SDI for each year, 1996–2005, for all Brazilian states

**Fig. 4:**
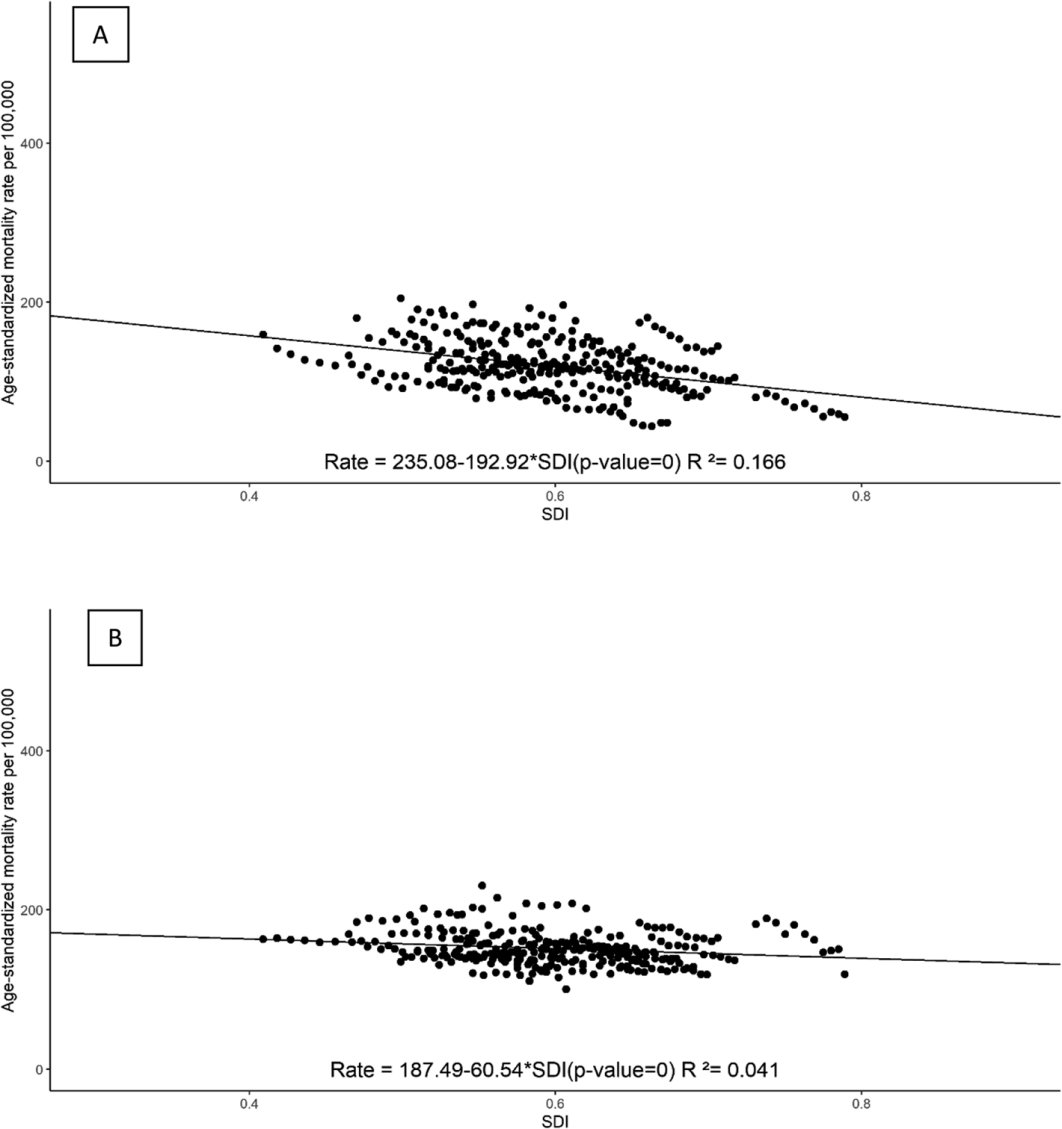
Age-standardized mortality rates for deaths coded to level 1 or 2 garbage codes (**a**) and to level 3 or 4 garbage codes (**b**) by SDI for each year, 2006–2016, for all Brazilian states

Figures 3 and 4 show the relationship between levels of SDI and rates of GCs coded into two groups, levels 1–2 and 3–4, for all states by year in two separate periods: 1996–2005 and 2006–2016. In the first period, there was an inverse relationship between rates of GC levels 1–2 and SDI, i.e., age-standardized rates of GC levels 1–2 decreased as the SDI increased for states. On the other hand, for GC levels 3–4, we found lower rates in less developed states. In 2006–2016, in contrast, the relationship between GCs and SDIs was inverse for both types of GCs.

We also evaluated the relationship between SDI and rate of deaths coded to GCs with linear regression models presented in Table 3 controlling for completeness. In 1996–2005, there was a significant inverse association between rates of GC levels 1–2 and SDI, and a significant direct association for levels 3–4, indicating that less developed states had higher rates of GC levels 1–2 and lower rates of GC levels 3–4. In 2006–2016, the coefficients were negative for the two GC groups, indicating that states with higher SDIs tend to have lower rates of all types of GCs. It is interesting to note here that although we had adjusted the GC rates for completeness, there was a significant direct association between completeness and rates of GC levels 3–4 in 1996–2005, i.e. states with lower completeness had lower rates of GC levels 3–4.

**Table 3:** Linear regression models of garbage codes (GCs) rates by SDI and completeness. Brazil, 1996–2016

	1996–2005	2006–2016
	Coefficients	
**Levels 1–2 GC**		
Intercept	691.71*	217.42*
SDI	− 912.03*	− 211.47*
Completeness	0.28	0.30
**Levels 3–4 GC**		
Intercept	− 25.61	210.46
SDI	146.04*	− 36.24
Completeness	118.71*	− 0.39

*p < 0.05

**Fig. 5:**
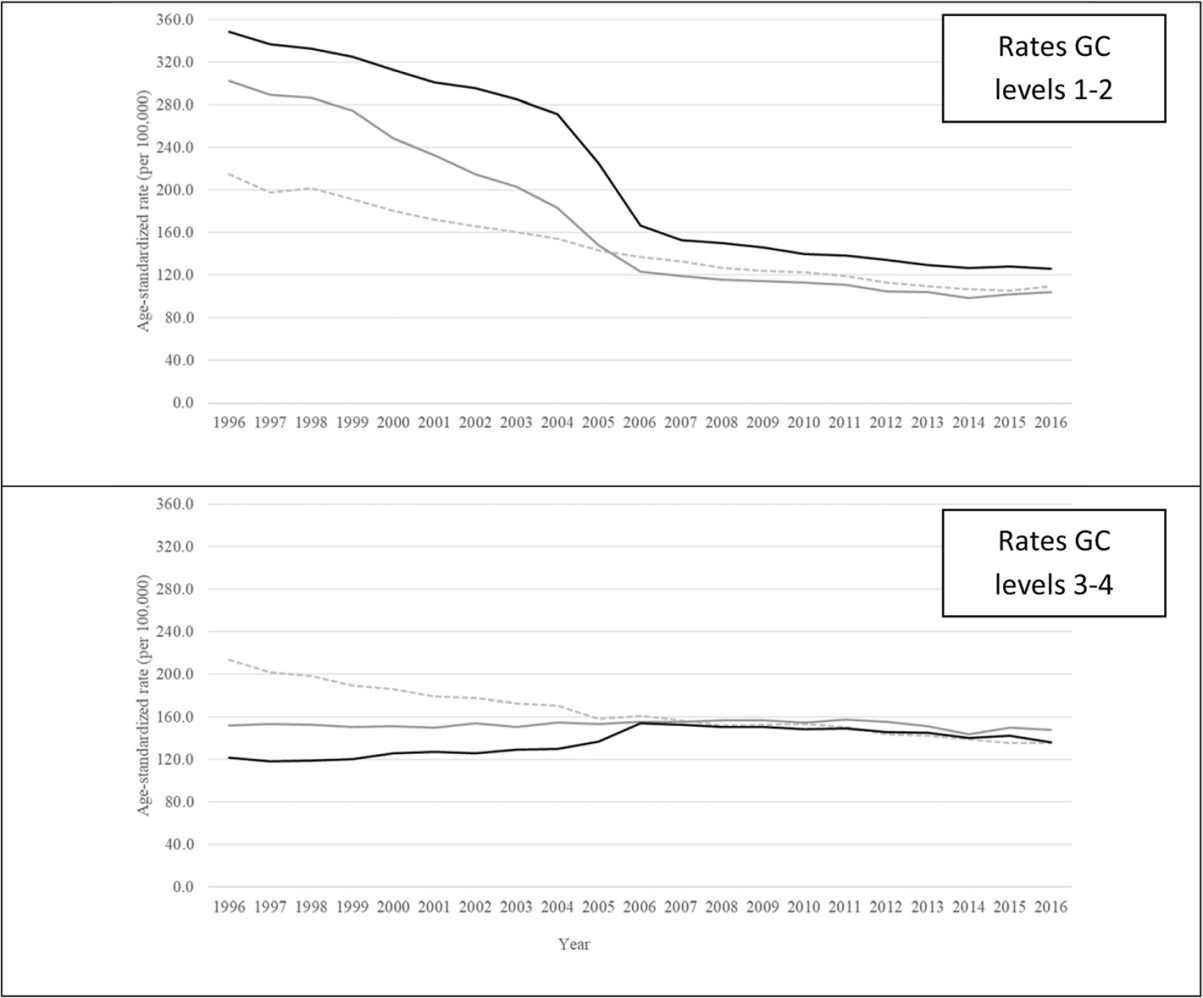
Age-standardized mortality rates of deaths coded to garbage code (GC) levels 1–2 (top panel) and 3–4 (bottom panel) according to SDI tertiles of Brazilian states from 1996 to 2016

Figure 5 presents temporal trends of rates of GCs in states grouped according the SDI in the initial analyzed year of 1996 into high (first tertile), medium (second tertile), and low (third tertile) level of development. Mortality rates due to GC levels 1–2 decreased over time in all SDI tertiles (Fig. 5—top panel), with the lowest SDI group having a sharp decline after 2004. On the other hand, for GC levels 3–4, this tertile had increased rates from 1996 to 2005, and decreasing rates occurred only in the higher developed states. All rates have been similar across tertiles in more recent years (Fig. 5—bottom panel). States in the highest tertile of SDI have had high completeness since 1996. On the other hand, states in the lowest tertile of SDI had completeness of death counts in the SIM of about 65% in 1996 (Supplementary file 3).

## Discussion

Estimated probabilities of having a GC as the cause of death decreased 40.4% from 1996 to 2016 in Brazil and the decrease was particularly important for the level 1 codes (58.4% over the period). The sharp decline presented by this group may be related to the investigation in hospitals and at home of an important part of GCs from this group, the R-codes, which has been performed by the Ministry of Health (MoH) since 2005 [5, 14]. States classified in low or medium SDI tertiles were responsible for the most important decline. Greater improvement in Brazil´s less developed states might be related to investments in the public health care system [15]. Although the relationship between SDI and rates was maintained in 2006–2016, rates of level 1–2 GCs in the three different state groups converged over time, indicating reduced inequalities in data quality due to major GCs. This reduction may have been related to the intervention of the MoH focused on poorer areas, and therefore leading to a reduction in those codes [6].

The decrease of major GCs occurred in almost all Brazilian states, and this encouraging result also indicates progress in strengthening cause-of-death statistics in the country over the past 20 years. Our findings are supported by the results of the classification system developed for the GBD2016 study—the GBD data quality rating. The calculation of this rating is based on the following variables: completeness of death registration, proportions of deaths not coded to GC levels 1 or 2, and fraction of deaths assigned to detailed GBD causes. The score for Brazil improved from 68.9 in 1995–1999 to 82.5 in 2010–2017, although it remained as a 4-star country in this 1 to 5 star classification. In the latter period, some high-income countries like Germany (score = 84.0), the Netherlands (83.3), and Japan (81.3) were also classified with 4 stars. On the other hand, in South America, Chile and Colombia were classified with 5 stars, but Uruguay, Paraguay, and Argentina were also classified with 4 stars [10].

Although with a decreasing trend, GCs still represent an important percentage of total deaths in the country, influencing the quality of mortality information. Despite the improvement in cause of death quality, R-codes and other ill-defined causes such as heart failure or septicemia, which contain no information of the underlying cause, remain frequent causes. High proportions of deaths due to these causes indicate not only worse quality of cause of death statistics, but also lack of medical care, especially among the economically disadvantaged population [16]. Interesting, proportions of pneumonia unspecified increased in 2016 for men and women, probably related to the observed increase over time in age-specific mortality rates from specific infections of the lower respiratory tract in the population aged 70 years or over [17].

The highest proportion of GCs occurred among elderly people, especially those over 80 years (49.6%) and were due mainly to level 4 GCs. Higher proportions of ill-defined causes among the elderly are probably due to the higher number of comorbidities, such as neoplasms, hypertension, diabetes, and other cardiovascular diseases, making it difficult to provide information on the underlying cause of death during the completion of the DC [18–20], even after the use of verbal autopsies [21]. On the other hand, children aged 1–14 years also had a high fraction of major GCs, more than 20%. High proportions of GCs in this age group, although in accordance with the results of Naghavi et al. [8] in other settings, are of concern as it is very important for prevention to correctly identify the leading causes of premature deaths.

Regarding the place of death, GCs were registered as the underlying cause of death in almost 50% of home deaths and in more than 38% of those which occurred in a hospital. Although previous studies in some Brazilian cities showed that ill-defined R codes were negatively associated with hospital deaths [22, 23], the percentage found for in-hospital deaths in this study can be considered relatively high. In hospital settings diagnostic procedures are more available, and we expected that better defined CODs would be declared much more frequently.

The highest proportion of GCs in home deaths may be due to difficulties of diagnosing or a lack of knowledge of the diagnosis by the physician. This can be explained by the fact that the certification of a home death is more difficult than deaths occurring inside hospitals due to the difficulty of defining a diagnosis for reasons already mentioned. The majority of home deaths in Brazil had medical assistance before death [24]. This finding reinforces the importance of continuing the investigation of household deaths until a culture valorizing the importance of the correct DC information is establishing among physicians.

Proportions of deaths coded with GCs were similar among categories of certifying physicians, although we had originally expected a smaller proportion to be present when attested by the attending physician, and by those from SVO and forensic institutes. But for major GCs, the proportion of GC level 2 in deaths certified by forensic institute physicians was higher than that found for other physician categories. These findings probably result from deaths from injuries whose intent remained undetermined despite being certified by a coroner.

In order to investigate the impact of socioeconomic conditions on rates of different types of GCs over time, we have presented three sets of calculations at the state level. First, we presented age-standardized rates by SDI in two periods, 1996–2005 and 2006–2016. These results indicate that while there has been a decline of GC levels 1–2 in both periods, mortality rates coded to GC levels 3–4 increased as SDI increased across states and time during 1996–2005 but decreased as SDI increased during 2006–2016. Second, we presented linear regression models in two separate periods for analyzing coefficients of SDIs and completeness related to GCs. The inclusion of completeness was intended to address whether this variable impacted on GC occurrence, as there is evidence of an association of improvements in completeness with declining percentages of deaths being reported as ill-defined [4]. Finally, extended temporal analysis of states classified in tertiles of development according to state of residence ranked by estimated SDIs in 1996 indicated substantial differences in performance between low and high SDI tertile states in 1996–2005, with the gap closing substantially in the 2006–2016 period.

Our results of an inverse association between levels of SDI and rates of levels 1–2 GCs in both periods are consistent with recent publications focused on ill-defined causes of death. Higher proportions of R-codes from chapter 18-ICD10 are more frequent among people who had a lower educational level or less access to health services [25–27]. On the other hand, the unexpected significant direct relationship between SDI and mortality rates due to GC levels 3–4 in 1996–2005 constitutes the most striking finding from our study. To our knowledge, this finding was not reported before in the literature. We also observed a significant direct relationship between completeness and proportions of GC levels 3–4 in the first period, i.e., these GCs were higher when completeness increased.

The GBD 2017 study aggregates locations into 5 SDI groupings generated by applying quintile cutoffs from the distribution of national-level SDI for countries with populations greater than 1 million in 2017 to estimates of SDI for all GBD locations. Brazilian states were classified into 3 of those groupings: low-middle SDI, middle SDI and high-middle SDI [10]. We adapted this procedure by applying tertiles cutoffs to estimates of all state-level SDI in Brazil in 1996 to better contextualize the Brazilian reality. When analyzing temporal changes of GC rates across those tertiles of SDI, we observed that the increase of rates of GC levels 3–4 were concentrated in the low SDI tertile states in 1996–2005, and this slight increase occurred simultaneously with the increase in completeness in this group of states, and greater decrease of levels 1–2. It should be due to expanded primary care, with better knowledge of patient problems prior to the admission that led to death [27].

We suppose that states with better completeness and socioeconomic standards have more hospitals, and physicians were able to fill out the DC with GC levels 3–4 such as pneumonia, diabetes, or stroke unspecified, instead of an R code from level 1. The growing prevalence in poorer areas of some of these GCs such as diabetes of unspecified type, with higher burden in the North and Northeast regions [28], could also have had contributed. Another hypothesis is related to the correction of undercount of deaths in this study, as this adjustment was based on the assumption of a similar COD distribution in deaths recorded and not recorded in SIM, which is probably not correct for some causes. Different distribution of causes among registered and missing deaths was observed in Minas Gerais State, Brazil, using the verbal autopsy technique [29]. Further detailed investigation of this aspect can help us to identify specific issues of this subject.

Overall, the predominance of level 4 GCs in the country in recent years is encouraging, as these causes have less impact in public health. According to Anaconda, a tool for checking the quality of mortality data, GC levels 1–3 are classified as higher severity unusable causes, given their higher risk of misguiding public policies to avoid premature deaths. Level 4 codes are not included in the broad category of unusable causes by this classification. Furthermore, level 4 GCs include codes that may require access to greater diagnostic sophistication and considerable equipment to accurately determine the COD [30].

This study has two major limitations. First, GC mortality is corrected for under-registration using the estimates of all-cause mortality from the GBD study, which can be imprecise [10]. Furthermore, as was previously stated, the correction for cause-specific rates was based on the assumption of a similar COD distribution in deaths recorded and not recorded in the SIM, which may not be the case [29]. Secondly, to analyze socioeconomic differences at the regional level, we used the sociodemographic index (SDI), a composite indicator proposed in more recent GBD studies, as a measure of socioeconomic deprivation. We chose the SDI over other deprivation measures as it is frequently used in various studies as an indicator of the socioeconomic level of countries and subnational divisions and uses similar component indicators for countries and states [10], which facilitates comparisons in the global context. Nevertheless, there are no set criteria for judging the quality of estimates of measures of deprivation, and hence it is difficult to assess whether the SDI is the best indicator. However, it summarizes three important dimensions of human development and is calculated in years from 1990 to 2017, according to methodological adjustments made for the GBD 2017 study [10].

The analysis plan developed here does not contemplate the potentially important socioeconomic differences by sex in completeness and trends concerning the quality of cause-of-death registry in Brazil, which therefore may also be considered a limitation in this study. Our objective, however, was only to visualize the socioeconomic differences without control for confounding factors such as gender and access to health services. Additionally, if we were to stratify the analysis by sex, we would end up producing a considerably more detailed analysis, longer and more complex, and thus turning more difficult the task of understanding the principal objective, the description of the burden of GCs in Brazil.

In summary, this study provides a more accurate understanding of trends and magnitude of mortality coded to garbage codes according to levels and the ranking of specific groups. This information is of importance for health policy in Brazil as the proportions found in 2016 were still large enough to impact regional inequalities in well-defined causes of death without a redistribution of GCs. Although the GBD study redistributes those GCs among specific causes, this redistribution is based on statistical methods and has some limitations [10, 31]. As better correction is provided by empirical data [32], this study might also contribute to the recent effort made by the Ministry of Health who in 2017 implemented important interventions in 60 municipalities throughout the country, as part of the Bloomberg Data for Health Initiative, in which hospital deaths with garbage codes were investigated extracting information from hospital records using standardized questionnaires, which then were reviewed by physicians in order to identify the underlying or specific causes. This initiative, reinforced by adequate physician training on the filling in of the DC, has significantly improved data from the SIM [33]. So, it is imperative to have a comprehensive knowledge of GC distribution in the country, therefore permitting further advances in this intervention and the subsequent empirical corrections.

## Conclusions

There has been a decreasing burden of deaths coded to GCs in Brazil. Analysis differentiated by types of GCs and level of development shows different temporal trends and substantial inequalities within the Brazilian population in 1996–2005. Decreasing inequalities in mortality coded to GCs over the period 2006–2016 and the predominance of less important GC level 4 in the country in 2016 indicate progress in strengthening cause-of-death statistics in Brazil over the past 20 years.

## Supplementary information

**Supplementary file 1:** Title: List of garbage codes mapped to the Global Burden of Disease 2017 cause list. Description: The source is: GBD 2017 Causes of Death Collaborators. Supplementary appendix 1. Supplement to: Global, regional, and national age-sex-specific mortality for 282 causes of death in 195 countries and territories, 1980–2017: a systematic analysis for the Global Burden of Disease Study 2017. Lancet. 2018; 392:1736–88.

**Supplementary file 2:** Title: Deaths classified as garbage codes according to selected variables in males and females. Brazil, 2016. Description: It shows the percentage of GC and non-GC coded deaths in males and females by age, place of death and certifying physician in 2016.

**Supplementary file 3:** Title: Completeness according to SDI tertiles of Brazilian states from 1996 to 2016.

## Data Availability

The dataset used and analyzed in the study is available from the Brazilian Ministry of Health (www.datasus.gov.br).

## Abbreviations

COD: Cause of death
DC: Death certificate
GBD: Global Burden of Disease
GC: Garbage code
ICD: International Classification of Disease and Related Health Problems
IHME: Institute for Health Metrics and Evaluation
SDI: Sociodemographic Index
SIM: Mortality Information System [Sistema de Informações sobre Mortalidade]
SVO: Death Investigation Service [Serviço de Verificação de Óbitos]
WHO: World Health Organization
