# Supplementary material for: Changes in the quality of cause-of-death statistics in Brazil: garbage codes among registered deaths in 1996–2016
Source: Popul Health Metr. 2020 Sep 30;18(Suppl 1):20. doi: 10.1186/s12963-020-00221-4 (PMC7526091; doi:10.1186/s12963-020-00221-4)
Supplement: Supplementary file 1 — Title: List of garbage codes mapped to the Global Burden of Disease 2017 cause list. Description: The source is: GBD 2017 Causes of Death Collaborators. Supplementary appendix 1. Supplement to: Global, regional, and national age-sex-specific mortality for 282 causes of death in 195 countries and territories, 1980–2017: a systematic analysis for the Global Burden of Disease Study 2017. Lancet. 2018; 392:1736–88. [file 12963_2020_221_MOESM1_ESM.docx]

**Supplementary file 1: List of garbage codes mapped to the Global Burden of Disease 2017 cause list**

| **Level** | **ICD10** |
| --- | --- |
| Garbage Codes  (Level 1)* | A31-A31.9, A40-A44.9, A48.0, A48.3, A49.0-A49.1, A59-A59.9, A71-A71.9, A74.0, B07-B07.9, B30-B30.9, B35-B46.9, B49-B49.9, B55.1-B55.2, B58-B59.9, B85-B85.4, B87-B88.9, B94.0, C46-C46.9, D50-D50.0, D50.9, D62-D63.0, D63.8-D64, D64.1-D65.9, D68, D69.9, D80-D84.9, D89.8-D89.9, E12-E15, E16, E50-E50.9, E64.1, E85.3-E87.6, E87.8-E87.9, F06.2-F06.4, F07.2, F09-F09.9, F17-F17.9, F20-F23.9, F25-F49, F51-F99.0, G06-G08.0, G32-G32.8, G43-G44.2, G44.4-G44.8, G47-G47.2, G47.4-G47.9, G50-G60.9, G62-G65.2, G80-G83.9, G89-G89.4, G91-G91.2, G91.4-G92.9, G93.1-G93.2, G93.4-G93.6, G98, G99-H05, H05.2-H69.9, H71-H99, I26-I26.9, I31.2-I31.4, I37.9, I46-I46.9, I50-I50.9, I51.7, I67.4, I76, I95-I95.1, I95.8-I95.9, J40-J40.9, J47-J47.9, J69-J69.9, J80-J80.9, J85-J85.3, J86-J86.9, J93-J93.1, J93.8-J93.9, J94.2, J96-J96.9, J98.1-J98.3, K00-K19, K30, K65-K66.1, K66.9, K70.4-K71.6, K71.8-K73.9, K75.0, L20-L30.9, L40-L50.9, L52-L54.8, L56-L56.2, L56.4-L56.5, L57-L57.9, L59-L68.9, L70-L76.8, L80-L87.9, L90-L92.9, L94-L96, L98.5-L99.8, M04, M10-M12.0, M12.2-M29, M37-M39, M43.2-M49, M49.2-M64, M65.1-M71, M71.2-M72.4, M72.8-M73, M73.8-M79.9, M83-M86.2, M86.5-M86.9, M87.2-M87.9, M89.1-M89.4, M90-M99.9, N17-N17.9, N19-N19.9, N32.1-N32.2, N32.8-N33.8, N35-N35.9, N37-N37.8, N39.3-N39.8, N42-N43.4, N44.1-N44.8, N46-N48.9, N50-N53.9, N61-N64.9, N82-N82.9, N91-N91.5, N95, N95.1-N95.9, N97-N97.9, R02-R02.9, R03.1, R07.0, R08-R09, R09.3, R11-R12.0, R14-R15.9, R19-R19.6, R19.8-R23, R23.1-R30.9, R32-R50.1, R50.8-R57.9, R58.0-R78, R78.6-R94.8, R96-R99.9, U05, U07-U81, U89.9-U99, X40-X44.9, X49-X49.9, Y10-Y14.9, Y16-Y19.9, Z00-Z15.8, Z17-unsp. |
| Garbage Codes (Level 2) | A14.9, A29-A30.9, A45-A45.9, A47-A48, A48.8-A49, A49.3-A49.9, A61-A62, A72-A73, A76, A97, B08-B09, B11-B14, B28-B29, B31-B32.4, B34-B34.9, B61-B62, B68-B68.9, B73-B74.2, B76-B76.9, B78-B81.8, B84, B92-B94, B94.8-B94.9, B95.6-B97.3, B97.7-B99.9, D59, D59.4, D59.8-D59.9, G44.3, G91.3, G93.0, G93.3, I10-I10.9, I15-I15.9, I27-I27.0, I27.2-I27.9, I28.9, I70-I70.1, I70.9, I74-I75.8, J81-J81.1, J90-J90.0, J94-J94.1, J94.8-J94.9, K92.0-K92.2, N70-N71.9, N73-N74.0, N74.2-N74.8, R03-R03.0, R04-R06.9, R09.0-R09.2, R09.8-R10.9, R13-R13.9, R16-R18.9, R23.0, R58, S00-T98.3, W47-W48, W63, W71-W72, W76-W76.9, W82, W95-W97, W98, X07, X55-X56, X59-X59.9, Y20-Y34.9, Y86-Y87, Y87.2, Y89, Y89.9-Y99.9 |
| Garbage Codes (Level 3) | A01, A49.2, A64-A64.0, A99-A99.0, B89, C14-C14.9, C26-C29, C35-C36, C39-C39.9, C42, C55-C55.9, C57.9, C59, C63.9, C68, C68.9, C75.9-C80.9, C87, C97-D00.0, D01, D01.4-D02, D02.4-D02.9, D07, D07.3, D07.6-D09, D09.1, D09.7, D09.9-D10, D10.9, D13, D13.9-D14, D14.4, D17-D21.9, D28, D28.9-D29, D29.9-D30, D30.9, D36.0, D36.9-D37.0, D37.6-D38, D38.6-D39.0, D39.7, D39.9-D40, D40.9-D41, D41.9, D44, D44.9, D48, D48.7-D49.1, D49.5, D49.7-D49.9, D54, D75.9, D79, D85, D87-D88, D90-D99, E07.8-E08.9, E17-E19, E34.9-E35.8, E37-E39, E47-E49., E62, E69, E87.7, E90-E998, F04-F06.1, F06.5-F07.0, F07.8-F08, F50, F50.8-F50.9, G09-G09.9, G15-G19, G21-G21.2, G21.4-G22.0, G27-G29, G33-G34, G38-G39., G42, G48-G49, G66-G69, G74-G79, G84-G88, G93, G93.8-G94.8, G96-G96.9, G98.0-G98.9, I00.0, I03-I04., I14-I14., I16-I19, I29-I29.9, I44-I45.9, I49-I49.9, I51, I51.6, I51.8-I59, I90-I94, I96-I96.9, I98.4-I98.8, I99-ID5.9, J02.9, J03.9, J04.3, J06, J06.9, J48-J59, J71-J79, J81.9, J83, J85.9, J87-J89, J90.9, J93.6, J97-J98.0, J98.4-J99.8, K31.9-K34, K39, K47-K49, K53-K54, K63-K63.4, K63.8-K63.9, K69, K75, K78-K79, K84, K87-K89, K92, K92.9-K93, K96-K99, L06-L07, L09, L15-L19, L31-L39, L69, L77-L79, N09, N13-N13.9, N24, N28.8-N28.9, N38, N39.9-N40.9, N54-N59, N66-N69, N78-N79, N84, N84.2-N86, N88-N90.9, N92-N94.9, N95.0, O08-O08.9, O17-O19, O27, O37-O39, O49-O59, O78-O79, O93-O95.9, P06, P16-P18, P30-P34.2, P40-P49, P62-P69, P73, P79, P82, P85-P89, P96.9-P99.9, Q08-Q10.3, Q19, Q29-Q29., Q36.0-Q36.9, Q46-Q49, Q88, Q89.9, Q94, Q99.9-R01.2, R07, R07.1-R07.9, R31-R31.9 |
| Garbage Codes (Level 4) | B54-B55, B55.9, B64, B82-B82.9, B83.9, G00, G00.9-G02.8, G03.9, I42-I42.0, I42.9, I51.5, I64-I64.9, I67, I67.8-I68, I68.8-I69, I69.4-I69.9, J07-J08, J15.9, J17-J19.6, J22-J29, J64-J64.9, P23, P23.5-P23.9, P37.3-P37.4, V87-V87.1, V87.4-V88.1, V88.4-V89.9, V99-V99.0, Y09-Y09.9, Y85-Y85.9 |
| Source: GBD 2017 Causes of Death Collaborators. Supplementary appendix 1. Supplement to: Global, regional, and national age-sex-specific mortality for 282 causes of death in 195 countries and territories, 1980–2017: a systematic analysis for the Global Burden of Disease Study 2017. Lancet. 2018; 392:1736–88. | |
| *Note: The following codes were classified in other levels: J47, K70.4-K70.9 considered as level3; E12-E15 considered as level 4; K73 considered as level 2. | |
|  | |
|  | |
