# Supplementary material for: Changes in the quality of cause-of-death statistics in Brazil: garbage codes among registered deaths in 1996–2016
Source: Popul Health Metr. 2020 Sep 30;18(Suppl 1):20. doi: 10.1186/s12963-020-00221-4 (PMC7526091; doi:10.1186/s12963-020-00221-4)
Supplement: Supplementary file 2 — Title: Deaths classified as garbage codes according to selected variables in males and females. Brazil, 2016. Description: It shows the percentage of GC and non-GC coded deaths in males and females by age, place of death and certifying physician in 2016. [file 12963_2020_221_MOESM2_ESM.docx]

Supplementary file 2. Deaths classified as garbage codes according to selected variables in males and females. Brazil, 2016

|  |  | Garbage Codes | | | | | | | | |  | Total deaths (%)** | |
| --- | --- | --- | --- | --- | --- | --- | --- | --- | --- | --- | --- | --- | --- |
|  |  | level 1 (%)* | | level 2 (%)* | | level 3 (%)* | | level 4 (%)* | | Total GC (%)* | |  |  |
|  |  | Male | Female | Male | Female | Male | Female | Male | Female | Male | Female | Male | Female |
| **Age** | < 01 | 6.2 | 5.8 | 1.0 | 0.8 | 3.0 | 3.0 | 4.2 | 4.3 | 14.4 | 13.9 | 20191(2.7) | 15969(2.8) |
|  | 1-4 | 20.7 | 19.6 | 3.2 | 3.6 | 2.2 | 3.0 | 11.5 | 14.6 | 37.7 | 40.8 | 3362(0.5) | 2849(0.5) |
|  | 5-9 | 18.2 | 19.3 | 3.8 | 3.0 | 2.1 | 3.1 | 9.3 | 9.9 | 33.4 | 35.3 | 1859(0.3) | 1438(0.3) |
|  | 10-14 | 15.0 | 20.1 | 4.1 | 2.9 | 1.9 | 2.2 | 7.4 | 8.1 | 28.3 | 33.5 | 3009(0.4) | 1867(0.3) |
|  | 15-19 | 6.4 | 16.3 | 4.2 | 3.7 | 0.5 | 2.3 | 6.0 | 9.0 | 17.1 | 31.3 | 17988(2.4) | 3797(0.7) |
|  | 20-29 | 6.8 | 13.2 | 4.4 | 3.7 | 0.9 | 2.5 | 7.1 | 8.8 | 19.1 | 28.2 | 45248(6.1) | 10393(1.8) |
|  | 30-39 | 10.1 | 11.9 | 4.6 | 3.5 | 1.9 | 3.5 | 8.6 | 9.0 | 25.3 | 27.9 | 46230(6.3) | 18625(3.3) |
|  | 40-49 | 12.3 | 11.7 | 4.7 | 3.8 | 3.2 | 4.5 | 10.9 | 10.9 | 31.2 | 30.9 | 60178(8.2) | 32465(5.7) |
|  | 50-59 | 12.5 | 11.3 | 4.5 | 3.8 | 4.0 | 5.0 | 13.0 | 13.4 | 34.0 | 33.5 | 99031(13.4) | 58755(10.3) |
|  | 60-69 | 12.2 | 11.7 | 4.2 | 4.1 | 3.9 | 4.5 | 16.1 | 17.7 | 36.5 | 38.0 | 132642(18.0) | 89089(15.6) |
|  | 70-79 | 12.9 | 13.3 | 4.3 | 4.7 | 3.7 | 3.9 | 19.7 | 21.9 | 40.6 | 43.8 | 143836(19.5) | 121362(21.2) |
|  | 80 e+ | 15.8 | 17.0 | 5.2 | 6.1 | 3.6 | 3.6 | 22.7 | 24.8 | 47.3 | 51.4 | 160703(21.8) | 215400(37.6) |
|  | Missing | 22.5 | 24.6 | 11.1 | 10.3 | 1.1 | 3.7 | 12.0 | 14.0 | 46.7 | 52.6 | 2565(0.3) | 350(0.1) |
|  | **Place of death*** | |  |  |  |  |  |  |  |  |  |  |  |
| **Place of death*** | Hospital/health facility | 11.0 | 11.9 | 3.7 | 3.8 | 3.8 | 4.2 | 17.9 | 20.6 | 36.4 | 40.5 | 504547(68.5) | 443219(77.4) |
|  | Home | 20.7 | 22.1 | 6.8 | 8.3 | 3.0 | 3.4 | 12.7 | 15.8 | 43.3 | 49.6 | 144820(19.7) | 111295(19.4) |
|  | Street | 4.8 | 8.5 | 4.3 | 5.2 | 0.3 | 0.8 | 8.6 | 14.1 | 17.9 | 28.6 | 52717(7.2) | 7161(1.3) |
|  | Others | 12.0 | 17.1 | 7.1 | 7.5 | 1.1 | 2.5 | 8.0 | 14.0 | 28.2 | 41.1 | 33714(4.6) | 10292(1.8) |
|  | Missing | 21.1 | 30.4 | 8.6 | 7.9 | 2.0 | 2.6 | 8.4 | 15.8 | 40.1 | 56.6 | 1044(0.1) | 392(0.1) |
|  | **Who signed the DC** | | |  |  |  |  |  |  |  |  |  |  |
| **Who signed the DC** | Assistant physician | 12.2 | 13.1 | 3.7 | 4.3 | 4.0 | 4.1 | 18.0 | 20.7 | 37.8 | 42.2 | 179036(24.3) | 164718(28.8) |
|  | Substitute physician | 12.2 | 12.6 | 3.2 | 3.5 | 4.3 | 4.4 | 18.3 | 20.2 | 38.1 | 40.8 | 149573(20.3) | 144007(25.2) |
|  | Forensic institute physician | 10.7 | 19.5 | 14.9 | 14.8 | 0.6 | 1.4 | 7.6 | 10.1 | 33.8 | 45.8 | 141860(19.3) | 33301(5.8) |
|  | SVO physician | 13.3 | 15.8 | 3.4 | 4.3 | 2.9 | 3.0 | 14.8 | 15.2 | 34.4 | 38.3 | 49001(6.7) | 37816(6.6) |
|  | Another physician | 18.2 | 18.3 | 4.8 | 5.3 | 4.1 | 4.3 | 16.7 | 19.5 | 43.8 | 47.4 | 142720(19.4) | 128461(22.4) |
|  | Missing | 26.1 | 26.3 | 5.2 | 5.5 | 3.5 | 3.8 | 15.8 | 19.0 | 50.6 | 54.6 | 74652(10.1) | 64056(11.2) |

*Fractions of each row total; ** Fractions of all deaths (n=736842-male and n=572359-female).
