# Supplementary material for: Changes in the quality of cause-of-death statistics in Brazil: garbage codes among registered deaths in 1996–2016
Source: Popul Health Metr. 2020 Sep 30;18(Suppl 1):20. doi: 10.1186/s12963-020-00221-4 (PMC7526091; doi:10.1186/s12963-020-00221-4)
Supplement: Supplementary file 3 — Title: Completeness according to SDI tertiles of Brazilian states from 1996 to 2016. [file 12963_2020_221_MOESM3_ESM.docx]

|  |
| --- |

**Supplementary file 3**. **Completeness according to SDI tertiles of Brazilian states from 1996 to 2016.**
